# Book Reviews

**Published:** 1917-03

**Authors:** 


### BOOK REVIEWS

Books mentioned may be inspected at and ordered through this office. So far as possible, books received in any month will be reviewed in the issue of the second month following. Pamphlets, quarterly and similar periodicals, reports, transactions, etc., will, as a rule, merely be mentioned.

**Freud's Theories of the Neuroses.** Dr. Eduard Hitschmann, Vienna, authorized translation by Charles Rockwell Payne, M. D., Moffatt, Yard & Co., N. Y., 257 pages, $2.00.

**The Neurotic Constitution.** Dr. Alfred Adler, Vienna, authorized translation by Bernard Gluck, M. D. and John E. Lind, M. D., Moffatt, Yard & Co., N. Y., 456 pages, $3.00.

These books deal with essentially the same subject, viewed from different angles. They discuss, the latter in greater variety and detail, the expressions of mental states in dreams, subconsciously, or in forms of sexuality, often perverted or inverted. While not in themselves controversial, they naturally tend to continue the controversy aroused as to the Freudian conceptions. As for other works along this subject, it seems to us that undue prominence is ascribed to the sexual factor, especially when it is interpreted from dreams, obsessions and tendencies that do not directly suggest it. It must, of course, be admitted that this factor is frequently the dominating one even in normal persons, more so in neurotics. Still, it may be questioned whether the sexual factor is necessarily exaggerated in neurotics.

**Federal Responsibility in the Solution of the Habit-Forming Drug Problem.** Charles B. Towns, M. D., N. Y. Pamphlet.

The U. S. Commissioner of Internal Revenue has proposed certain amendments to the Harrison Law which may be summed up as applying a stamp to the taxation and control of narcotics and as making the law conform more closely to the general provisions of internal revenue statutes. The author divides the users of narcotics into hopeless, bona fide addicts who, after registration for purposes of identification, should be allowed access to such drugs, without extra expense or subterfuge; persons who have acquired the habit in the course of a painful disease no longer existing but who can stop it without definite treatment; underworld addicts, for whom some international system of control of the supply is necessary. The pamphlet deserves careful study.

**A Manual of Nervous Diseases,** by Irving J. Spear, M. D., Professor of Neurology at the University of Maryland, Baltimore. 12mo of 660 pages with 169 illustrations. Philadelphia and London: W. B. Saunders Company. 1916. Cloth, $2.75 net.

The size of the pages (5¾x3¾ net) somewhat handicaps the author in showing tables and illustrations; otherwise the frequent use of these methods of teaching, is excellent. The descriptions are clear and the conception good. For instance, note how the whole matter of apoplexy, etc., is clarified by the mere title: "Rupture or Occlusion of the Cerebral Arteries." Bone lesions, both as causes and results of nervous conditions, are of course elucidated by Roentgenology. A considerable group of heretofore puzzling and unclassifiable conditions is now logically considered as symptomatic of disease of the ductless glands. The infectious element in the etiology of various nervous diseases is mentioned only incidentally and it seems that the author is somewhat sceptic on this point. For example, he estimates that "fully 40% of the acute choreas are caused by the absorption of toxic substances which are formed as the result of bacterial activity," whereas many writers convey the impression that most such manifestations are directly infectious. The recent claim of a bacterium for epilepsy is ignored and tetanus is discussed with achondroplasia, as an unclassified disorder, hydrophobia being merely mentioned in the differential diagnosis. This method discussion is sound. Too often a discussion of nervous diseases is a treatise on syphilis, tuberculosis and infections in general, with only incidental allusion to the distinctly nervous manifestations, and the reader receives an exaggerated impression of the role of bacteria.

---

**Care of Patients** undergoing Gynecologic and Abdominal Procedures, before, during, and after operation by E. E. Montgomery, M. D., Professor of Gynecology in Jefferson Medical College, Philadelphia. 12mo of 149 pages with 61 illustrations. Philadelphia and London: W. B. Saunders Company. 1916. Cloth, $1.25 net.

This work was prepared during the convalescence of the author from an operation—a lesson in industry that many of us may take to heart. Like many other useful books, it is somewhat difficult to classify. It is not strictly a treatise for nurses nor an operating room manual while, of course, it makes no pretense to being a work on general surgery. It deals with the details of attention to the patient, provision of instruments and dressings and the very important part of surgery after general scientific knowledge and technical skill are taken for granted. It is a work that appeals especially to surgical nurses, internes, surgical assistants and those ambitious to develop that degree of surgical skill which not only gives to the term success, a statistic meaning but which interprets it as the survival and health of the patient.

---

**A Text-Book on the Practice of Gynecology.** For Practitioners and Students. By W. Easterly Ashton, M. D., LL.D., Professor of Gynecology in Graduate School of Medicine of the University of Pennsylvania. Sixth Edition, Thoroughly Revised. Octavo of 1097 pages with 1052 original line drawings. Philadelphia and London: W. B. Saunders Company, 1916. Cloth, $6.50 net; Half Morocco, $8.00 net.

The first 150 pages are devoted to general considerations, such as the technic of examinations, causes of disease peculiar to women, history taking, methods of examining the abdomen and rectum, use of X-rays, and includes several chapters that may be considered the medical aspects of gynaecologic diagnosis and treatment, as microscopic and bacteriologic examinations and that of the blood, hydrotherapy, diet, exercise and saline injections. The further arrangement of the work is, so far as practicable, by organs, as the vulva, vagina, uterus, tubes, ovaries, urinary organs, etc., and by such topics as ectopic gestation, suppuration of the pelvic connective tisue, menstrual disorders, etc. Thus surgery is not unduly emphasized nor taken out of its proper relations to other means of treatment, although fully discussed. The author is conservative but in the true conservative sense. The number of editions through which the work has passed not only attests its excellence but has given the opportunity for still greater excellence.

---

**A Treatise on Diseases of the Skin.** For the use of advanced Students and Practitioners. By Henry Stelwagon, M. D., Ph. D., Professor of Dermatology, Jefferson Medical College, Philadelphia. Eighth edition, thoroughly revised. Octavo of 1309 pages, with 356 text-illustrations, and 33 full-page colored and half-tone plates. Philadelphia and London: W. B. Saunders Company, 1916. Cloth, $6.50 net; Half Morocco, $8.00 net.

Thorough text books on this subject have become quite well standardized as to arrangement, and this work has become standard as an authority, One notes, however, a gradual and now quite marked departure from the artificial system of classifying skin diseases by superficial lesions, and a gratifying diminution in those conditions which are not well understood as to general nature. Syphilis remains an important part of dermatology is becoming a true specialty and not merely a combination with venereal diseases.

---

**Evolution Proving Immortality.** John O. Yeiser, Omaha. Published by National Magazine Assn., Omaha. 200 pages, illustrated, paper covers, $1.50.

While for obvious reasons, it is unwise for a medical journal to attempt a critical review of the arguments adduced, it may be said that the book is interesting and in line with the present interests of the public.

---

**The Practical Medicine Series.** Charles L. Mix, A. M., M. D., General Editor. The Year Book Publishers, 327 S. La Salle St., Chicago. Series 1916, 10 volumes, $10.

Vol. 9, Skin and Venereal Diseases, Oliver S. Ormsby and James Herbert Mitchell, editors, 238 pages, $1.35 separately.

Vol. 10, Nervous and Mental Diseases, Hugh T. Patrick, Peter Bassoe and Lewis J. Pollock, editors, 232 pages, $1.35 separately.

These volumes complete the 1916 series which fully maintains the standard of efficiency previously set for this comprehensive, specialized review of periodical medical literature.

---

**Fern Notes.** Oliver Atkins Farwell, Illustrated pamphlet reprinted from 18th Annual Report of Mich. Acad. of Science, Dec. 1916.

---

**Medical Clinics of Chicago.** Bi-monthly, W. B. Saunders Co., Philadelphia, $8 per year. Jan. 1917, Vol. 2, No. 4.

---

**Clinical Gynecology.** By James C. Wood, A. M., M. D., F. A. C. S., etc. 236 pages. Cloth, $2.00 net. Philadelphia, Boericke & Tafel, 1917.

While avowedly a homoeopathic text book, the author emphasizes the importance of a broad basis of general information. The author, though a surgeon, does not discuss operative technic although he has an interesting chapter on postoperative surgical treatment. On the other hand, the work is not a medical gynaecology in the extreme sense; only in that operative measures are merely suggested in general terms.

Considerable space is given to border-land conditions, as gastro-enteric auto-intoxication, mucous entero-colitis, gastric and duodenal ulcer, thyroid diseases, etc.

---

**Hygiene in Mexico,** A Study of Sanitary and Engineering Problems, Alberto J. Pani, C. E. City of Mexico; translated by Ernest L. de Gogorza; published by G. P. Putnam's Sons, N. Y., 206 pages, $1.50.

The author states that his sole purpose was to expose one of the most nefarious inheritances from the past and that the entire proceeds of the Spanish edition were placed at the disposal of the People's University of Mexico, to further hygienic teaching. A chart shows the total mortality of Mexico City in 1911 as a trifle over 42: 1000, the next highest rates for cities between 400,000 and 700,000 being for Cairo, Egypt, 40, and Madras, British India, nearly 40. Next in order come Madrid, Marseilles, Dublin and Rome, about 23. The average for European cities is 17.53, for American cities 16.1 (really a better rate for the former, considering the direct influence of adult immigration in adding to population without the high death rate incident to the breeding process). It is surprising to note the minimum rates: Amsterdam and Rotterdam, 12.4, Frankfort-am-Main 12.1, and Smyrna, 10.83. The relation of illiteracy to high mortality is shown in another chart, though the relation is not at all exact. The book is certainly timely.

